# Intrauterine chilled saline instillation reduces endometrial impairment on MRI after ultrasound-guided percutaneous microwave ablation of uterine adenomyosis

**DOI:** 10.1186/s13244-024-01707-7

**Published:** 2024-06-05

**Authors:** Hui-Li Zhang, Er-Ya Deng, Jing-E Zhu, Jia-Xin Li, Le Fu, Li-Ping Sun, Cheng-Zhong Peng, Xiao-Long Li, Song-Yuan Yu, Hui-Xiong Xu

**Affiliations:** 1 Department of Medical Ultrasound, Center of Minimally Invasive Treatment for Tumor, Shanghai Tenth People’s Hospital, Ultrasound Research and Education Institute, Clinical Research Center for Interventional Medicine, School of Medicine, Tongji University, Shanghai, China; 2 Shanghai Engineering Research Center of Ultrasound Diagnosis and Treatment, Shanghai, China; 3 Department of Radiology, Shanghai First Maternity and Infant Hospital, School of Medicine, Tongji University, Shanghai, China; 4 Department of Ultrasound, Zhongshan Hospital, Institute of Ultrasound in Medicine and Engineering, Fudan University, Shanghai, China

**Keywords:** Adenomyosis, Percutaneous microwave ablation, MRI, Endometrial impairment

## Abstract

**Objective:**

To investigate whether intrauterine chilled saline can reduce endometrial impairment during US-guided percutaneous microwave ablation (PMWA) of adenomyosis.

**Methods:**

An open-label, randomized trial was conducted with sixty symptomatic adenomyosis patients who were randomly assigned (1:1) to receive PMWA treatment assisted by intrauterine saline instillation (study group) or traditional PMWA treatment alone (control group). The primary endpoint was endometrial perfusion impairment grade on post-ablation contrast-enhanced MRI. The secondary endpoints were endometrial dehydration grade, ablation rate, and intra-ablation discomfort.

**Results:**

The baseline characteristics of the two groups were similar. The incidence rates of endometrial perfusion impairment on MRI in the study and control groups were 6.7% (2/30) and 46.7% (14/30), respectively (*p* < 0.001). There were 28 (93.3%), 2 (6.7%), 0, and 0 patients in the study group and 16 (53.3%), 7 (23.3%), 5 (16.7%), and 2 (6.7%) in the control group (*p* < 0.001) who had grade 0, 1, 2, and 3 perfusion impairment, respectively. Additionally, there were 27 (90%), 3 (10%), and 0 patients in the study group and 19 (63.3%), 10 (33.3%), and 1 (3.3%) in the control group who had grade 0, 1, and 2 endometrial dehydration (*p* = 0.01). The ablation rates achieved in the study and control groups were 93.3 ± 17% (range: 69.2–139.6%) and 99.7 ± 15.7% (range: 71.5–129.8%), and they were not significantly different (*p* = 0.14). No significant difference was found in the intra-ablation discomfort.

**Conclusion:**

Intrauterine chilled saline can effectively reduce endometrial impairment after PMWA treatment for adenomyosis.

**Critical relevance statement:**

This trial demonstrated that the instillation of intrauterine chilled saline reduced endometrial impairment on MRI during PMWA of adenomyosis. This approach allows more precise and safe ablation in clinical practice.

**Key Points:**

- Endometrial impairment occurs in the PMWA treatment of adenomyosis.
- Intrauterine chilled saline can reduce endometrial impairment during PMWA for adenomyosis.
- An intrauterine catheter is a practical endometrial protecting method during thermal ablation.

**Trial registration:**

Chinese Clinical Trial Registry, ChiCTR2100053582. Registered 24 November 2021, www.chictr.org.cn/showproj.html?proj=141090.

**Graphical Abstract:**

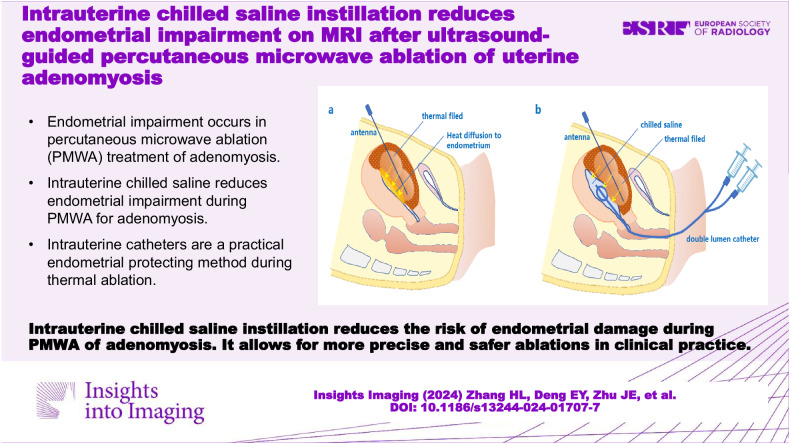

## Introduction

Adenomyosis is a common but burdensome condition in reproductive-aged women [1], with a prevalence of 20.9% in patients who undergo ultrasound (US) screening in a gynecology clinic [2]. Adenomyosis can cause progressive worsening of dysmenorrhea, heavy menstrual bleeding, anemia, and infertility, and has a substantial impact on patients’ physical and mental health. Currently, the treatment of symptomatic adenomyosis consists mainly of medication, surgery, and conservative interventional therapies [3]. For patients who are unsuitable for surgery or long-term medication therapy, interventional therapy, including uterine artery embolization, MR-guided or US-guided high-intensity focused ultrasound (HIFU), transcervical radiofrequency ablation, and percutaneous microwave ablation (PMWA), is an alternative treatment [4–6].

Among treatment options, US-guided PMWA has attracted increasing attention in recent years. As a new minimally invasive treatment for uterine leiomyomas and adenomyosis, PMWA has several advantages over other interventional therapies [6–9], such as high efficacy and efficiency, low invasiveness, rare major complications, and no radiation exposure. Additionally, PMWA can effectively relieve adenomyosis-related symptoms and improve quality of life [10–12] without significantly affecting ovarian function [13]. However, its application for patients who wish to maintain fertility is still controversial because of limited clinical evidence regarding fertility outcomes [3, 14] and potential endometrial thermal damage after treatment [7, 15]. For example, endometrial perfusion impairment after thermal ablation was reported in 43.6% of patients with submucosal fibroids [16]. Severe bilateral endometrial damage can cause significant endometrial atrophy, fibrosis, scarring, partial adhesion, or obliteration of the uterine cavity [17], which is reportedly associated with hypomenorrhea, infertility, and increased pregnancy complications [18, 19]. Among patients without fertility requirements, thermal damage to the bilateral endometrium is also related to an increased risk of intrauterine adhesion (IUA) [20, 21], with a high risk of developing Asherman’s syndrome in the long term. Therefore, a practical and effective method for endometrial protection needs to be developed.

**Fig. 1:**
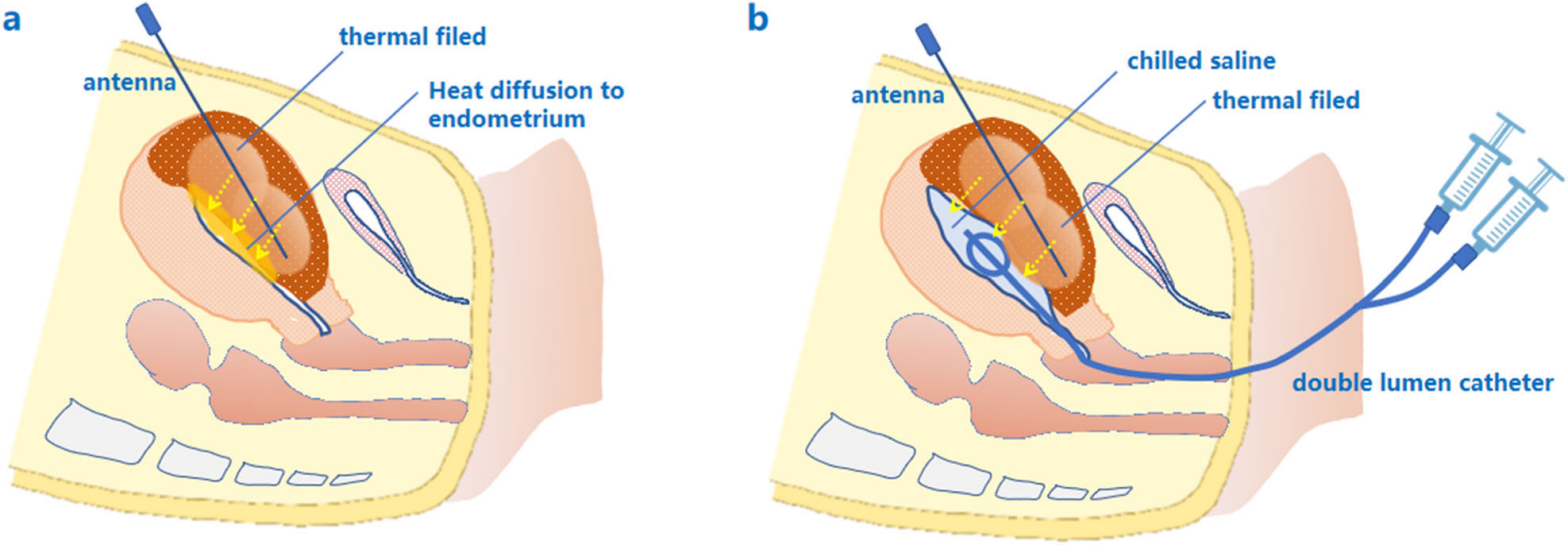
**a**, **b** Schematic drawings of intrauterine cooling by injection of chilled saline via a catheter. In traditional PMWA, the heat diffused from the thermal field can increase the intrauterine temperature (arrows), and cause damage to the endometrium and the junction zone of the contralateral wall (**a**). By injecting chilled saline into the uterine cavity via a double-lumen catheter (**b**), the endometrium around the ablation zone can be effectively cooled down

In a previous study, percutaneous intrauterine instillation of chilled saline was reported to be effective in protecting the endometrium during microwave ablation (MWA) of type 1–3 leiomyomas [22]. However, the procedure of percutaneous puncture with a core needle itself is quite operator-dependent and invasive to a certain extent, which limits its application in clinical practice. Intrauterine saline instillation through a soft urinary catheter is easy to perform (Fig. 1), almost noninvasive, and commonly used in the practice of uterine ablation for patients who are sexually experienced. However, no study has focused on the prevention of thermal damage to the endometrium after PMWA in adenomyosis patients. Herein, this study aimed to investigate whether intrauterine saline cooling with a urinary catheter is effective in protecting the endometrium during PMWA treatment of adenomyosis.

## Methods

### Study design and patient selection

This was an open-label, single-center, prospective, randomized trial that was approved by the institutional ethics committee of Shanghai Tenth Peoples’ Hospital (No. 21XJS39). The study was registered at www.chictr.org.cn, with the research number ChiCTR2100053582, and conducted following the Declaration of Helsinki. Patients who met the following criteria were enrolled and excluded.

The inclusion criteria were as follows: (1) premenopausal women with symptomatic adenomyosis (≥ 18 years); (2) both US- and magnetic resonance imaging (MRI)-diagnosed adenomyosis; (3) childbirth completed and had no fertility desire; (4) refused other treatments and had no contraindications to PMWA (e.g., severe cardiopulmonary dysfunction, coagulation dysfunction, and mental illness); and (5) willing to participate in the study and sign the informed consent forms. The exclusion criteria were (1) adenomyosis with bilateral wall involvement (*n* = 14); (2) concomitant submucosal leiomyomas (*n* = 14); (3) inability to undergo MRI examination due to metal implants in the body or contrast agent allergy (*n* = 3); (4) pre-ablation MRI revealing IUA or uterine deformities, indicating difficulty in placing a catheter into the uterine cavity (*n* = 4); (5) endometrial atrophy and thinness (thickness < 3 mm) leading to difficulty in assessing the degree of endometrial impairment (*n* = 1); or (6) planned to undergo transvaginal MWA treatment (*n* = 2). Finally, 60 patients were included in this study.

**Fig. 2:**
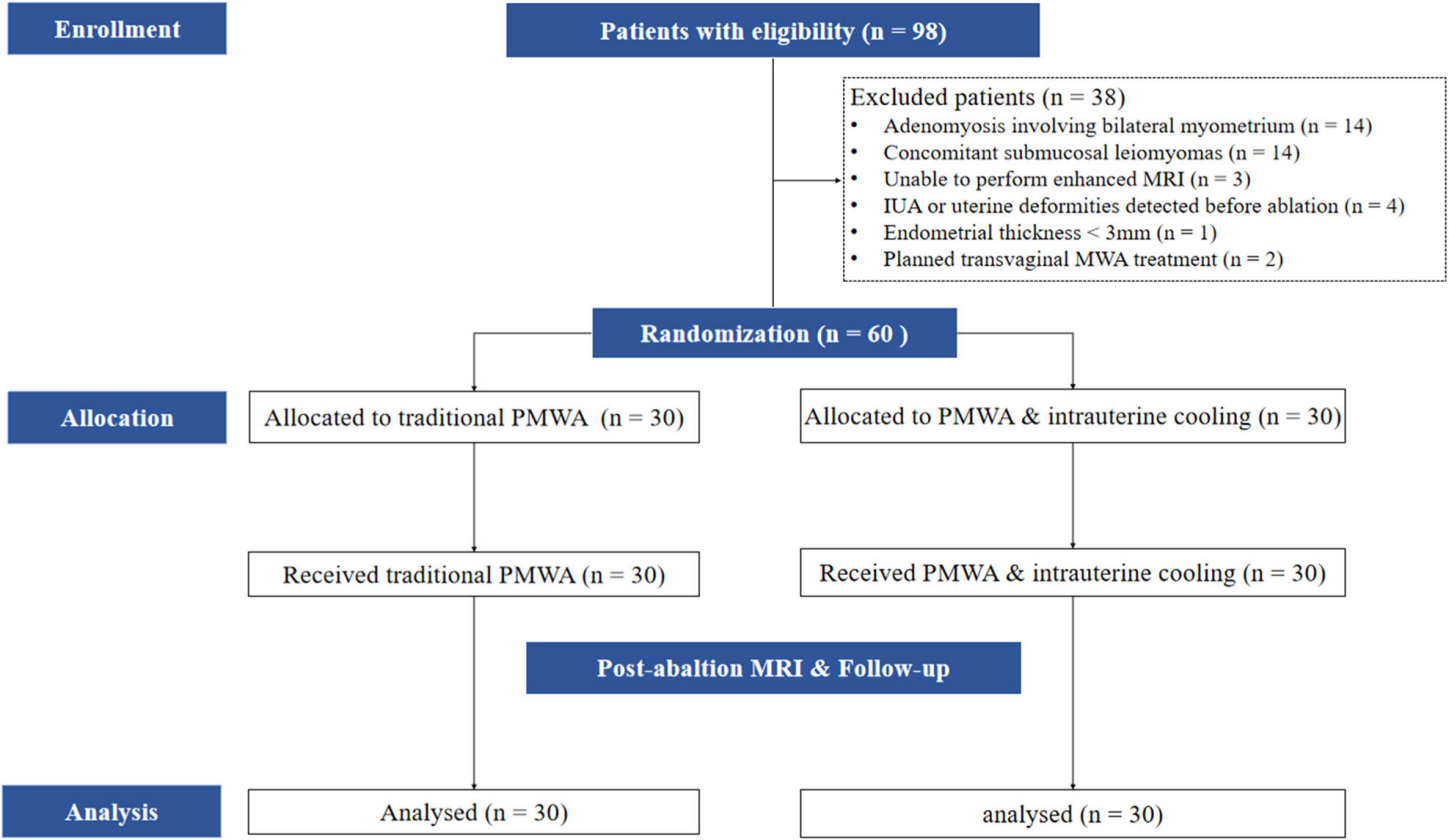
Flowchart of the randomized controlled trial

**Fig. 3:**
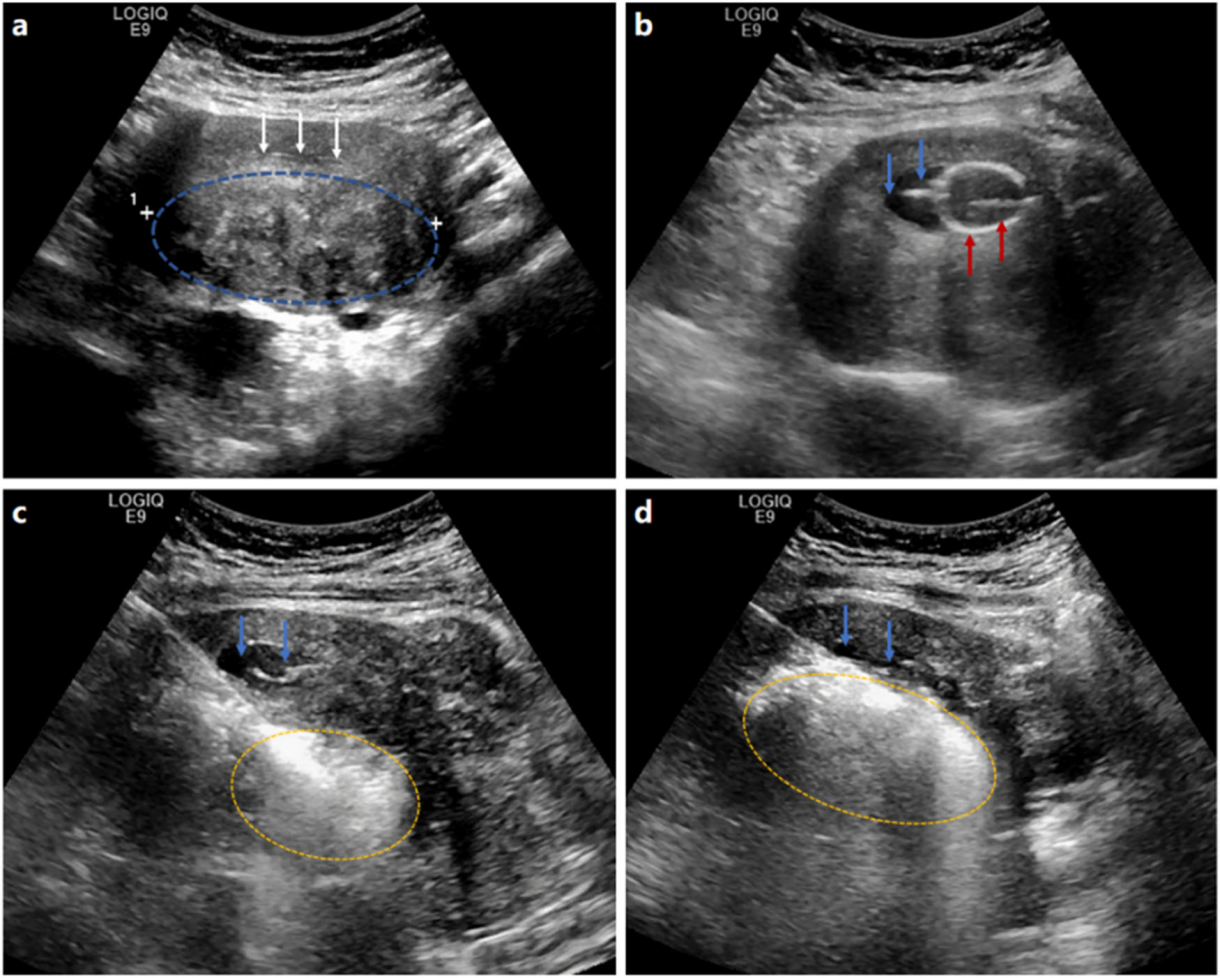
**a**–**d** A case of adenomyosis treated by PMWA assisted by intrauterine chilled saline. Before treatment, US (transverse section) reveals type IV adenomyosis (blue circle) located on the posterior wall (**a**). The endometrium is thin (3 mm), which leads to a blurred display of uterine cavity lines on B-mode US (white arrows). After injecting 5 mL of saline through a urinary catheter (red arrows), the uterine cavity lines separate from each other and display clearly (blue arrows) on B-mode US (**b**). Then the deepest part of the lesion (yellow circle) is ablated at first (**c**), with the uterine cavity displayed clearly (blue arrows). When ablating adenomyosis near the uterine cavity (yellow circle), chilled saline is injected into the uterine cavity slowly (blue arrows) and continuously to maintain a low temperature (**d**)

**Fig. 4:**
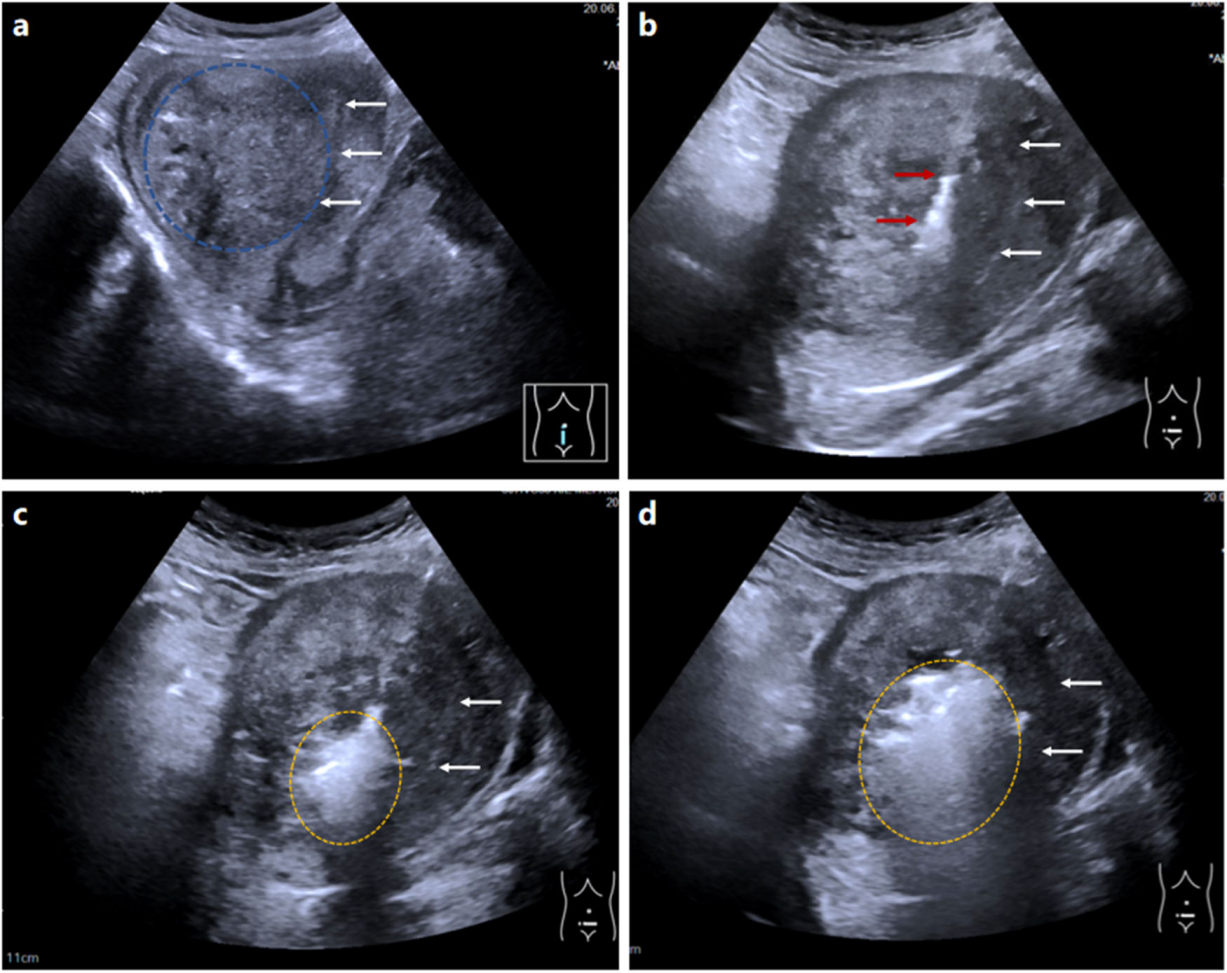
**a**–**d** A case of adenomyosis treated by PMWA only. Before treatment, US (sagittal section) reveals type IV adenomyosis (blue circle) located on the posterior wall (**a**). The endometrium (white arrows) is thin (4 mm), which leads to blurred cavity lines on B-mode US, and the antenna (red arrows) is inserted into the lesion 2 cm from it (**b**). The ablation starts at the deepest part (yellow circle) (**c**) when the cavity line still can be displayed at this point (white arrows). When the main part of the lesion is ablated (yellow circle), the endometrial line turns invisible due to gas interference (white arrows), making it impossible to determine whether heat has spread to the uterine cavity (**d**)

The study process is presented in Fig. 2. From December 2021 to July 2023, ninety-eight eligible patients who wished to undergo PMWA treatment were enrolled prospectively. All patients were informed of the benefits and potential risks of PMWA treatment and intrauterine saline installation, and written informed consent was obtained from each patient. After exclusions of patients who met the exclusion criteria, sixty patients were included and randomly assigned at a 1:1 ratio by a computer-generated randomization list into two groups. The study group received PMWA treatment assisted by intrauterine chilled saline (Fig. 3), while the control group received traditional PMWA treatment without intrauterine chilled saline (Fig. 4). As an open-label design was necessary, the allocation information was available to the clinical staff members, each of whom were blinded to the post-ablation evaluation personnel to avoid subjective factors that might bias the research results.

### Baseline information collection

The baseline information of all participants, including age, body mass index (BMI), complaints, medication history, and pregnancy and delivery history, among other variables, was collected before treatment. Symptoms were evaluated by an interventional US radiologist who specializes in gynecological interventional treatment. A pictorial blood loss assessment chart (PBAC) recommended by the Chinese expert consensus on the chemical ablation of uterine fibroids [23] was used to quantify the menstrual bleeding volume. Visual analog scale (VAS) scores ranging from 0 (no pain at all) to 10 (severe pain equivalent to childbirth pain) were used to quantify the severity of pain. The symptom severity score (SSS) and health-related quality of life (HRQOL) scale were used to quantify the severity of symptoms and patients’ quality of life [24].

### Pre-ablation evaluation

All patients underwent systematic examinations, including laboratory tests, chest X-ray, electrocardiography, pelvic US, and contrast-enhanced MRI, within 2 days before treatment. To rule out pelvic infection and early pregnancy, all patients underwent human chorionic gonadotropin tests and microbial testing of vaginal secretions. Pelvic US examination was performed with a US scanner (LOGIQ E9 system; GE Healthcare, Milwaukee, WI, USA) equipped with a 1–6 MHz convex array probe and 3–12 MHz intracavity probe. In total, 2 mL SonoVue^TM^ (Bracco Suisse SA, Geneva, Switzerland) was injected intravenously for contrast-enhanced ultrasound (CEUS) imaging before ablation. MRI was performed on a 1.5-T MR scanner (Magnetom Verio, Siemens, Germany) with a gradient slew rate of 200 mT/ms and gradient strength of 40 mT/m. Gadopentetate dimeglumine (Magnevist, Bayer Schering, Germany) was used as the contrast agent for contrast-enhanced T1-weighted imaging (CE-T1WI).

Based on pre-ablation T2-weighted imaging (T2WI), adenomyosis was classified into four types according to Kish’s study [25]: type I (intrinsic), internal adenomyosis surrounded by a layer of normal outer myometrium; type II (extrinsic), external adenomyosis with involvement of the outer myometrium only; type III (intramural), intramural isolated adenomyosis without connection to the endometrium or serosa; and type IV (mixed), adenomyosis penetrating the entire myometrium. The baseline volume of the uterine corpus and adenomyosis was measured on T2WI, with the ellipsoid analog volume formula (*V* = π/6 × height × length × width) adopted in the calculation.

### Pre-ablation preparation and intrauterine catheterization

All patients took oral laxatives to clear their intestines one day before treatment. Twenty minutes before ablation, all patients were given conscious sedation and analgesia, namely, slow intravenous injection of midazolam (0.1 mg) to relieve anxiety and fear and intravenous pumping of fentanyl (0.02 mg/kg) to alleviate pain. A portable electrocardiogram monitor was used to monitor the patient’s heart rate, blood pressure, respiration rate, and oxygen saturation throughout the entire procedure. With the patient placed in the lithotomy position, the external genitalia were disinfected. The cervical os was exposed with a speculum inserted into the vagina and then cleaned with povidone-iodine solution. An 8 F urinary catheter was inserted through the cervical os and into the cervical canal very gently with air evacuated. The catheter balloon was then inflated with 2–3 mL of saline, and the speculum was subsequently removed carefully. Finally, a 50 mL syringe filled with chilled sterile saline was attached to the catheter, which was prepared for intrauterine instillation during subsequent ablation therapy.

### US-guided PMWA treatment

A monopolar water-cooling MWA applicator (Microwave Ablation system MTI-5A; Nanjing Great Wall Medical Equipment Co. Ltd., Nanjing, China) with a power setting of 60 W was used. Correspondingly, an 18-cm long, 14-G microwave antenna (XR-A2018W; Nanjing Great Wall Medical Equipment Co. Ltd., Nanjing, China) was used. PMWA therapy was performed by two senior interventional US radiologists with more than 5 years of experience with the uterine ablation procedure. The operation process was standardized according to the recommendations of the Chinese expert consensus on MWA of adenomyosis [26], including disinfection and draping, local anesthesia, artificial ascites injection, US-guided biopsy, PMWA, and intra-ablation CEUS.

The patient was placed in the supine position, and the skin of the lower abdomen was disinfected. After administration of local anesthesia, 400–600 mL of warm sterilized saline was instilled into the pelvic cavity around the uterus to protect the surrounding organs. Next, a routine free-hand biopsy was performed under US guidance with an 18-G biopsy core needle (Bard Magnum Biopsy Instrument; Covington, GA, USA) to rule out potential malignancy. The puncture site was selected randomly to avoid large blood vessels. Subsequently, PMWA was performed with an output power of 60 W using the “moving-shot” technique under the guidance of transabdominal US. For patients with adenomyosis located at the posterior wall of a retroverted or retroflexed uterus, Yu’s uteropexy was applied to change the uterine position to avoid damage to the anterior endometrium [11].

To facilitate the precise placement of the microwave antenna, 5 mL of saline was injected into the endometrial cavity to visualize the spatial relationships among the antenna, adenomyosis, and endometrium (Fig. 3a, b). First, the antenna was inserted into the deepest part of the adenomyotic tissue 2 cm away from the serosa, and then microwave radiation was applied. Once the vaporization reaction was induced by heat, a hyperechoic cloud was generated and gradually spread out on B-mode US (Fig. 3c), allowing the ablation zone to be monitored in real time. A safe distance between the edge of the ablation zone and the serosa was maintained at least 5 mm to prevent thermal damage to the surrounding pelvic organs. When the region of adenomyosis near the endometrium (yellow circle) was ablated, chilled saline was slowly and continuously infused into the uterine cavity to maintain a low temperature (Fig. 3d). The amount of saline injected varied depending on the distance of the uterus (aimed to separate the uterine cavity lines by ≥ 5 mm) and patient tolerance. Generally, the total amount of saline instillation was controlled at ≤ 50 mL for each patient. If any pain occurred, the severity of the pain was evaluated and recorded by a dedicated US radiologist using a VAS, as was the pain induced by ablation. Finally, after the hyperechoic cloud covered the entire lesion on B-mode US, the ablation was stopped. After several minutes, intraoperative CEUS was performed to evaluate the instant local treatment response to PMWA, and supplementary ablation was performed if deemed necessary. The ablation was terminated when no enhancement was observed in the target lesion. At the same time, the extent of intra-ablation discomfort was recorded.

### Endometrial impairment evaluation

**Fig. 5:**
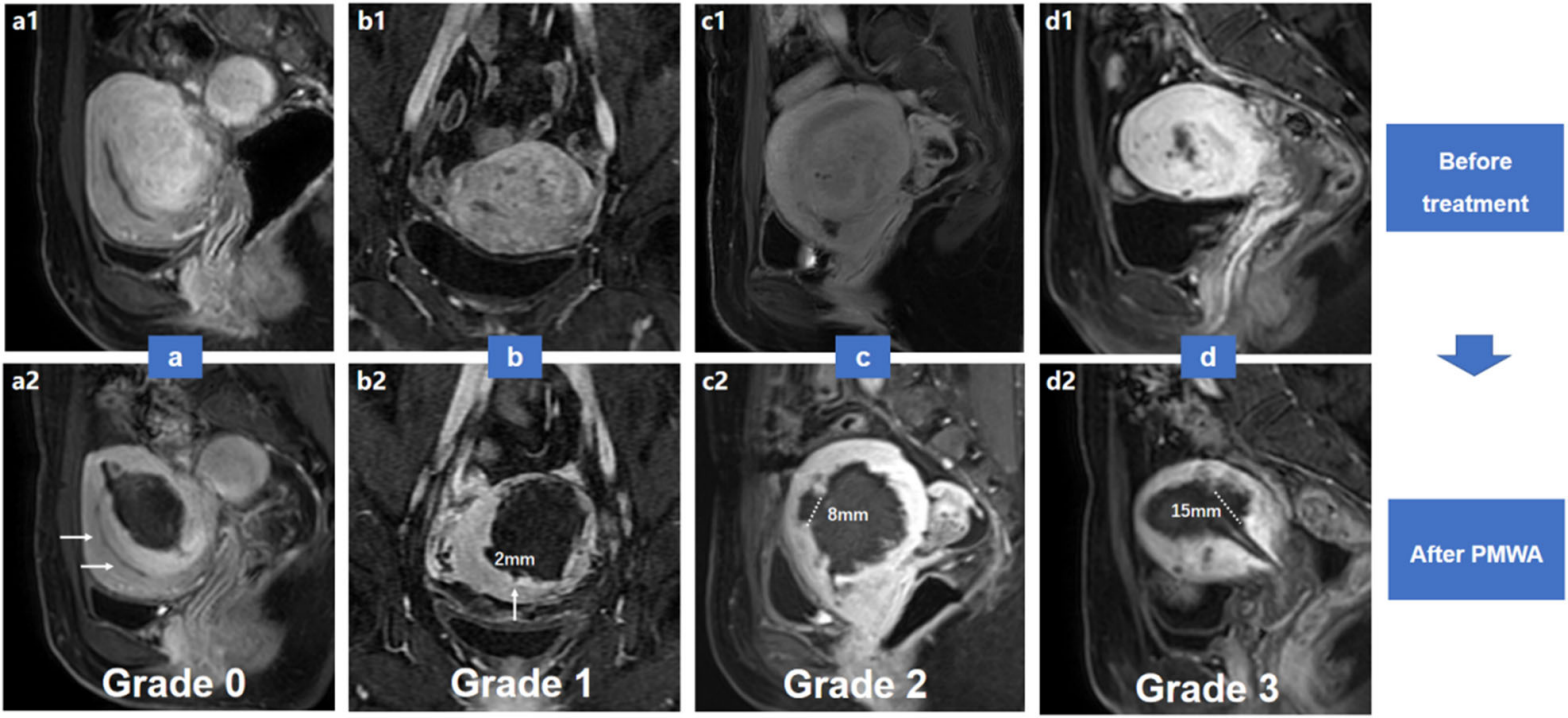
**a**–**d** Grading the perfusion impairment of the contralateral endometrium. Grade 0, pre-ablation CE-T1WI (**a1**) reveals uniform enhancement of the basal layer of endometrium, post-ablation CE-T1WI (**a2**) shows normal endometrium with similar enhancement (white arrows). Grade 1, pre-ablation CE-T1WI (**b1**) reveals enhanced bilateral endometrium, and post-ablation MRI (**b2**) shows pin-point enhancement discontinuity measured 2 mm (white arrow) on the endometrium of the anterior wall. Grade 2, pre-ablation CE-T1WI (**c1**) reveals type IV adenomyosis on the posterior wall, post-ablation CE-T1WI (**c3**) shows moderate enhancement discontinuity measuring 8 mm (dotted line) on the endometrium and junction zone of the anterior wall. Grade 3, pre-ablation CE-T1WI (**d1**) reveals adenomyosis on the anterior wall, while post-ablation CE-T1WI (**d2**) shows severe enhancement discontinuity measuring 15 mm (dotted line) on the endometrium and junction zone of the posterior wall

**Fig. 6:**
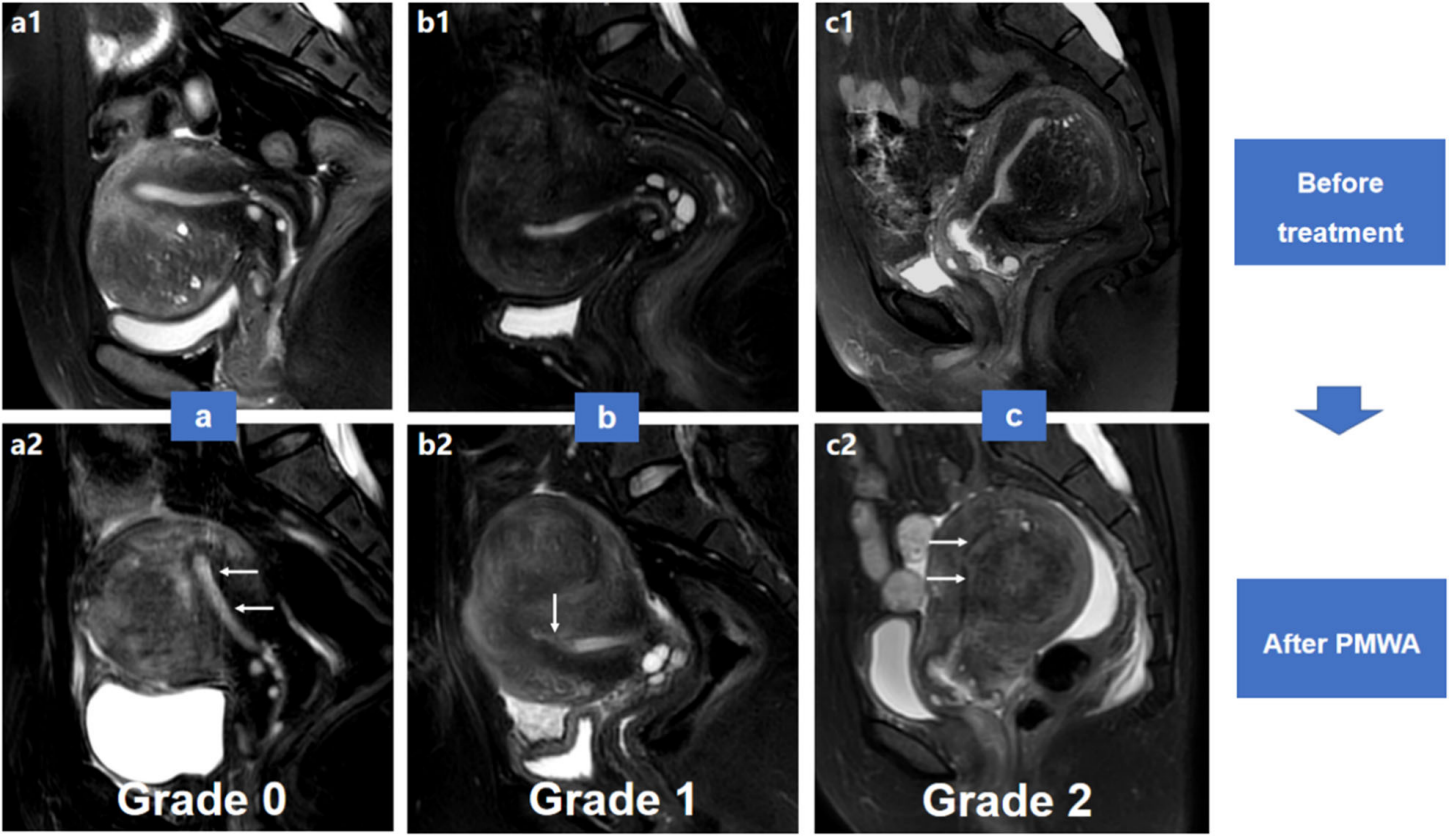
**a**–**c** Grading of the endometrial signal intensity impairment on T2WI. Grade 0, both pre-ablation (**a1**) and post-ablation (**a2**) sagittal fat-suppressed T2WI demonstrate hyper-intense endometrium (arrows). Grade 1, pre-ablation T2WI shows hyper-intense endometrium (**b1**), but post-ablation T2WI (**b2**) reveals heterogeneous endometrium with partial low T2 signal intensity (arrow), indicating mild endometrial dehydration and necrosis. Grade 2, almost the whole endometrium (arrows) demonstrates hypo-intensity after treatment (**c2**), indicating severe endometrial dehydration and necrosis

Contrast-enhanced MRI was performed within 2 days after treatment to investigate thermal damage to the endometrium. The assessment was carried out by two senior radiologists, who were blinded to the group allocation information. If a consensus was not reached, the final result was determined after discussion. Based on the enhancement defect on CE-T1WI, the thermal damage to the perfusion of the basal layer of the endometrium on the opposite side of the adenomyotic region was evaluated. In one patient whose fundus and posterior wall were affected, endometrial impairment in the anterior wall was assessed, and in another patient whose left wall and fundus were affected, endometrial impairment in the right wall was assessed. According to Kim’s study [16], endometrial perfusion impairment was divided into four levels: grade 0 (Fig. 5a), normal endometrium; grade 1 (Fig. 5b), mild, pinpoint discontinuity with a size ≤ 3 mm; grade 2 (Fig. 5c), moderate, discontinuity ≤ 1 cm, but > 3 mm; and grade 3 (Fig. 5d), severe, discontinuity > 1 cm. Based on the endometrial signal intensity (SI) displayed on T2WI after ablation, the severity of endometrial dehydration was evaluated and divided into three levels: grade 0 (Fig. 6a), normal high-signal endometrium; grade 1 (Fig. 6b), mild or moderate, with part of the endometrium showing hypointensity; and grade 2 (Fig. 6c), severe, with almost the whole endometrium showing hypointensity.

### Statistical analysis

The minimum sample size was calculated as indicated at http://powerandsamplesize.com. The intervention was considered effective if the incidence of endometrial impairment decreased by 0.35 in the study group. A sample of 26 participants per group provided at least 80% power with a 0.05 two-sided significance. Considering that some patients may have been excluded, which resulted in sample dropout (assuming ≤ 15%), the target number of enrolled patients was expanded to 31 per group.

SPSS 25.0 (IBM, Chicago, USA) was used for the statistical analysis. The distribution of the continuous data was tested by the Shapiro‒Wilk normality test. Normally distributed data are expressed as the mean ± SD, and nonnormally distributed data are expressed as the median (P25–P75). A *t* test was used to compare continuous variables with a normal distribution (age, BMI, and ablation rate), while the Wilcoxon signed-rank test was used to compare continuous data with a skewed distribution (VAS, PBAC, SSS, uterine volume, and adenomyosis volume). The Kruskal‒Wallis test was used to compare the ordered class variables (endometrial impairment grading results). The chi-square test or Fisher’s test was used to compare other categorical variables (uterine position, adenomyosis location, MRI classification, incidence of intraoperative discomforts). Statistical significance was defined as a *p* value < 0.05.

## Results

### Patient characteristics

The baseline characteristics of the patients in the two groups are summarized in Table 1. There were no significant differences in age, BMI, gravity, parity, baseline hemoglobin, or cancer antigen 125 level between the study and control groups. There was no significant difference in the proportions of patients in terms of uterine position, MRI type, or location of adenomyosis. In addition, no difference was observed between the two groups in terms of the quality-of-life score or severity-of-symptoms score, including the menstrual pain score, SSS, HRQOL, and PBAC.

**Table 1:** Comparison of the baseline characteristics between the two groups

Variables	Study group, (*n* = 30)	Control group, (*n* = 30)	*p* value
Age	42.8 ± 5.5	42.4 ± 6.8	0.82
BMI (kg/m^2^)	23.1 ± 3.5	23.2 ± 3.7	0.97
Gravidity	3 (2–4)	2 (0.8–3)	0.71
Parity	1 (0–1)	1 (1–1)	0.8
Hemoglobin (g/L)	76.7 (53.9–107.5)	94 (56.3–200)	0.59
Cancer antigen 125 (U/mL)	118.6 ± 28	116.2 ± 15.2	0.68
VAS	109 (89–117.3)	117.5 (106.8–129)	1
PBAC	179.5 (137.5–224.3)	133.5 (85–268.8)	0.07
SSS	36 (21.1–50)	26.6 (15.6–43.8)	0.59
HRQOL	52.4 ± 21	59.6 ± 23.8	0.21
Uterus			
Volume (mL)	271.5 (198.5–377)	212.9 (149–279.1)	0.07
Position			1
Anteverted	8	7	
Anteflexed	6	6	
Intermediate	6	7	
Retroverted	2	2	
Retroflexed	8	8	
Adenomyosis			
Volume (mL)	77.7 (50.5–100.7)	53.9 (40–88.2)	0.39
MRI classification			0.76
I	3	2	
II	5	7	
III	0	0	
IV	22	21	
Location			0.39
Anterior	9	7	
Posterior	20	20	
Lateral	1	1	
Fundal and posterior/lateral	0	2	

*BMI* body mass index

### Patient tolerance to treatment

A urinary catheter was successfully inserted into the uterine cavity through the vagina for all patients in the study group. During ablation of the adenomyosis near the endometrial cavity, chilled saline was slowly infused into the cavity, and the water flowed out through the vagina smoothly. There was no case of balloon rupture or spontaneous detachment during the treatment. During the process of the instillation of chilled saline into the uterine cavity, three patients reported mild lower abdominal pain (VAS score of 2–5), which improved after the amount of saline was reduced. Two patients reported significant lower abdominal pain (VAS scores of 7 and 8), and the US revealed left corneal adhesion in one patient and bilateral fallopian tube obstruction in another patient. The pain was also relieved after the amount of saline in the uterine cavity and catheter balloon was reduced and after analgesics were administered. None of the other 25 patients reported any discomfort.

Intraoperative discomfort included lower abdominal pain and nausea. The incidences of intra-ablation pain were 43.3% (13/30) and 63.3% (19/30) in the study group and control group, respectively (*p* = 0.12). The pain was effectively relieved in all patients after the intravenous addition of fentanyl (0.005 mg/kg). In addition, 2 and 1 patients in the study and control groups, respectively, reported nausea (*p* = 1), which was relieved after intravenous administration of palonosetron. No other discomfort was reported.

### Post-ablation evaluation results

The ablation rates of the study and control groups were 93.3 ± 17% (range: 69.2–139.6%) and 99.7 ± 15.7% (range: 71.5–129.8%), respectively, and no difference was found between them (*p* = 0.14). The results of the assessment of thermal damage to the endometrium and the SI are summarized in Tables 2 and 3.

**Table 2:** Comparison of endometrial perfusion impairment on the opposite wall

Variables	Study group, (*n* = 30)	Control group, (*n* = 30)	*p* value
Grade 0 (normal endometrium)	28	16	< 0.001
Grade 1 (pin-point discontinuity)	2	7	
Grade 2 (size of discontinuity ≤ 1 cm, but > 3 mm)	0	5	
Grade 3 (size of discontinuity > 1 cm)	0	2

**Table 3:** Comparison of endometrial T2 SI impairment between the two groups

Variables	Study group, (*n* = 30)	Control group, (*n* = 30)	*p* value
Grade 0 (hyper-intense)	27	19	0.01
Grade 1 (partial hypo-intense)	3	10	
Grade 2 (hypo-intense in the whole endometrium)	0	1	

*SI* signal intensity

According to the CE-T1WI results, the incidence of endometrial perfusion impairment on the side opposite to the site of adenomyosis in the study and control groups was 6.7% (2/30) and 46.7% (14/30), respectively (*p* < 0.001). As shown in Table 2, 28 (93.3%), 2 (6.7%), 0, and 0 patients had grade 0, 1, 2, and 3 endometrial perfusion impairment, respectively, in the study group, and 16 (53.3%), 7 (23.3%), 5 (16.7%), and 2 (6.7%) patients had grade 0, 1, 2, and 3 endometrial perfusion impairment, respectively, in the control group. No patients in the study group had grade 2 or 3 enhancement impairment. A significant difference was found in the severity of endometrial perfusion impairment between the two groups (*p* < 0.001). As shown in Table 3, 27 (90%), 3 (10%), and 0 patients, respectively, had grade 0, 1, and 2 endometrial dehydration in the study group, and 19 (63.3%), 10 (33.3%), and 1 (3.3%), respectively, had grade 0, 1, and 2 endometrial dehydration in the control group. Notably, no patients in the study group developed grade 2 endometrial SI impairment. The difference in the severity of endometrial SI impairment was also significant (*p* = 0.01). In general, compared with those in the control group, a significant decrease in the incidence of endometrial perfusion impairment (from 46.7% to 6.7%) and dehydration (from 36.7% to 10%) was observed in the study group.

The incidence of endometrial perfusion impairment or dehydration in patients with each adenomyosis type on MRI is summarized in Table 4. Moderate or severe (grade 2 or 3) endometrial perfusion impairment was observed only in type IV adenomyosis, with an incidence of 16.3% (7/43), but the difference in the incidence between different adenomyosis types was not significant (*p* = 0.25). Moreover, no difference was observed in the incidence of endometrial dehydration between different adenomyosis subtypes (*p* = 0.25).

**Table 4:** Comparison of endometrial impairment between three different adenomyosis types

Endometrial perfusion impairment	Type I, (*n* = 5)	Type II, (*n* = 12)	Type IV, (*n* = 43)	*p* value
Grade 0	4	11	30	0.25
Grade 1	1	1	6	
Grade 2	0	0	5	
Grade 3	0	0	2	

Endometrial dehydration	Type I, (*n* = 5)	Type II, (*n* = 12)	Type IV, (*n* = 43)	*p* value
Grade 0	3	11	32	0.25
Grade 1	1	1	11	
Grade 2	1	0	0	

## Discussion

US-guided PMWA has emerged as a new minimally invasive treatment for symptomatic adenomyosis, but its application for patients with a desire for fertility preservation is still controversial, partly because of the risk of endometrial thermal damage [14, 15, 21]. This study confirmed that intrauterine saline cooling via a urinary catheter was an effective and safe method for reducing endometrial impairment both in T2 SI and on MRI. This prospective randomized controlled study provides the highest level of evidence for the clinical application value of this method in the future. This was the first highlight of this study.

The study of endometrial impairment caused by thermal ablation was first conducted in patients with submucosal leiomyomas after HIFU treatment by Kim et al in 2017 [16], with the severity of damage graded based on the enhancement defect displayed on post-ablation MRI. Zhang et al reported that endometrial damage was common in the affected uterine wall of adenomyosis patients, with a percentage of 100% in patients with type IV adenomyosis and 60% in patients with type II adenomyosis [15]. The ablation area and percentage of the endometrial basal layer were associated with outcomes in patients with adenomyosis-induced abnormal uterine bleeding (AUB-A). In our center, for AUB-A patients without fertility desire, the endometrium adjacent to the adenomyosis site was usually ablated at the same time for good long-term outcomes, regardless of the MRI classification. In this study, 83.3% (10/12) of the type II adenomyosis patients had menorrhagia, and 66.7% of them had a history of secondary anemia. This contributed to the finding of no difference in the incidence of endometrial impairment among patients with different adenomyosis subtypes.

Among patients who do not seek fertility preservation, ablation of the bilateral endometrium is associated with a high risk of IUA. McCausland et al reported that partial or unilateral endometrial ablation with the opposite endometrium preserved did not cause IUA [20]. In other words, the protection of the endometrium on the wall opposite to that of the adenomyotic tissue would be key to reducing the risk of IUA after PMWA treatment. In this study, patients with type IV adenomyosis had a risk of significant endometrial perfusion impairment on the opposite side (16.3%), which revealed a certain risk of post-ablation IUA and suggested substantial clinical significance in protecting the endometrium in these patients. Therefore, we focused on the protection of the opposite endometrium in patients who did not wish to preserve their fertility. The primary endpoint was set as endometrial perfusion impairment on the opposite wall after the PMWA treatment of adenomyosis in patients with unilateral wall involvement. The incidence of endometrial perfusion impairment on the side opposite to that side of the adenomyotic tissue decreased from 46.7% (14/30) to 6.7% (2/30) after the assistance of intrauterine saline cooling.

Recently, percutaneous intrauterine chilled saline instillation with an 18-G puncture needle was reported to effectively reduce damage to the endometrium covering type 1–3 leiomyomas during US-guided MWA treatment [22]. However, the procedure of percutaneous puncture into the uterus itself is invasive and highly operator-dependent, with a risk of intraoperative bleeding. In addition, the uterus can move during ablation; therefore, there is a risk of scuffing the uterine cavity with the needle tip because it is metallic and sharp. It is generally considered the second-line endometrial protection method, and is usually applied to virgins without transvaginal operation routes in clinical practice. The most commonly used method for patients with vaginal access is to insert a soft, heat-resistant, rubber catheter into the uterine cavity through the cervical canal. It is mostly noninvasive, easy to perform, inexpensive, and unaffected by uterine movement. With the exciting results obtained in this study, it is promising for wide use in clinical practice in the future.

The hyaluronic acid gel has been reported to be the most effective approach for preventing moderate or severe adhesion formation after hysteroscopic adhesiolysis [27] and abortion [28], which leads to a high pregnancy rate. However, it is currently very expensive in China, and its viscosity causes the circulating fluidity to be inferior to that of sterile saline. In contrast to hysteroscopic surgery, the heat diffused from the thermal field to the endometrium needs to be carried outward through the vagina, indicating that liquids with good fluidity are more suitable for thermal ablation. Therefore, a small amount of chilled saline was used in this study following the steps of a saline infusion sonohysterography examination [29].

In this study, four patients were excluded due to MRI findings of uterine adhesion and septal uterus before treatment. Afterward, intrauterine catheterization was performed successfully in all the patients in the study group. It was promising that pre-ablation MRI was also useful for screening patients who were suitable for intrauterine saline cooling with a urinary catheter. In terms of patient tolerance, only two patients experienced moderate or severe pain (VAS score > 5) during saline instillation. Pain caused by increased intrauterine pressure was considered because significant adhesion of the left uterine horn and bilateral fallopian tube blockage were found. The pain was relieved after analgesia and the instillation of the indicated amount of saline. The intra-ablation discomforts included mainly lower abdominal pain and nausea in this study, and no significant difference was observed regarding their incidence. This means that the addition of intrauterine saline did not increase the risk of adverse intra-ablation events.

The second highlight of this study was that, in addition to perfusion damage, the change in the SI of the endometrium on T2WI was also evaluated as one of the second endpoints. The normal endometrium usually exhibits uniform hyperintensity on T2WI throughout different menstrual phases in premenopausal women [30]. Low SI on T2WI can have several causes, including tissue with low water content and/or low proton density, such as smooth muscle, fibrous tissue, or heavily calcified tissue [31]. Fresh blood clots can also appear as focal areas of low SI on MRI. In this study, the endometrium of each participant showed a high SI on T2WI before treatment, and thermal damage was the primary cause of the low-SI area after ablation, which indicated that endometrial dehydration occurred to different extents. This study revealed that after the adoption of intrauterine saline cooling, the percentage of patients with no endometrial dehydration increased from 63.3% to 90%. This provided solid evidence that intrauterine saline cooling helped reduce thermal damage to the endometrial cavity. It was quite inspiring that this method is promising for reducing endometrial damage and improving fertility outcomes in young patients who wished to preserve their fertility or had unplanned pregnancies after PMWA treatment. However, this finding needs to be confirmed through a new prospective study focused on the fertility outcomes of adenomyosis patients with a long follow-up period.

This study had several limitations. First, this was a single-center study with a small sample size. Although this was a randomized controlled study, the results still require external validation through additional multicenter studies. Second, post-ablation MRI was performed 2 days after treatment, and hematocele, exudation, and tissue edema in the uterine cavity may affect the accuracy of the endometrial impairment assessment. However, the final results were produced upon the agreement of two senior radiologists, so potential bias was minimized. Third, none of the patients underwent hysteroscopy after treatment, and endometrial impairment was assessed by MRI. As there was no case of Asherman’s syndrome or AUB during the posttreatment follow-up in this population, subsequent hysteroscopy examination was considered unnecessary. Finally, we did not include the follow-up results for endometrial recovery on MRI. A previous study revealed that there was significant repair of the endometrium after 3 months of HIFU of submucosal fibroids [32]. The repair status of the impaired endometrium after PMWA treatment in adenomyosis patients will be investigated in future studies.

## Conclusions

In summary, intrauterine chilled saline with a double-lumen catheter helped reduce endometrial impairment after PMWA treatment for adenomyosis.

## Data Availability

The data supporting this study’s findings are available from the corresponding authors under reasonable request.

## Abbreviations

AUB-A: Adenomyosis-induced abnormal uterine bleeding
BMI: Body mass index
CE-T1WI: Contrast-enhanced T1-weighted imaging
CEUS: Contrast-enhanced ultrasound
HIFU: High-intensity focused ultrasound
HRQOL: Health-related quality of life
IUA: Intrauterine adhesion
PBAC: Pictorial blood loss assessment chart
PMWA: Percutaneous microwave ablation
SI: Signal intensity
SSS: Symptom severity score
T2WI: T2-weighted imaging
VAS: Visual analog scale
